# Hybrid argon pasma coagulation-assisted mucosal ablation in the management of refractory gastroesophageal reflux disease

**DOI:** 10.1055/a-2229-4443

**Published:** 2024-01-23

**Authors:** Yun Bao, Silin Huang, Qiuping Qiu, Suhuan Liao, Xiaoping Hong, Miao He, Weiguang Qiao

**Affiliations:** 1 Department of Gastroenterology, South China Hospital, Medical School, Shenzhen University, Shenzhen, China; 2 Department of Gastroenterology, Southern Medical University, Nanfang Hospital, Guangzhou, China

A 59-year-old woman was admitted to our hospital for the management of refractory gastroesophageal reflux disease (GERD). Gastroscopy, ascertaining the presence of GERD, revealed a manifestation classified as LA-C, accompanied by a conspicuous abnormality in the gastroesophageal flap valve, graded as Type III ( Fig. 1 ). Dynamic reflux monitoring unveiled an acid exposure time of 32%, accompanied by a DeMeester score of 144.9.

**Fig. 1:**
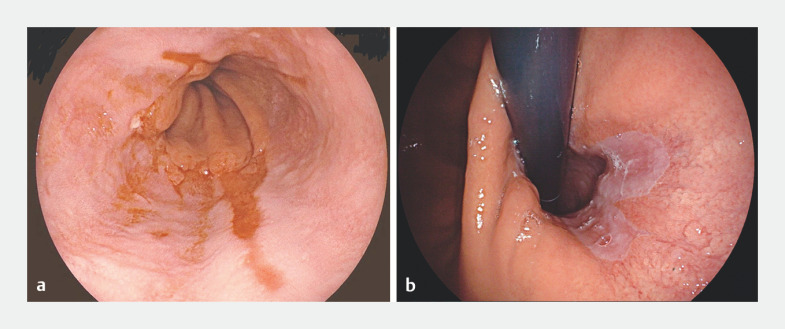
**a** The preoperative examination gastroscopy revealed reflux esophagitis LA-C. **b** It was accompanied by a conspicuous abnormality in the gastroesophageal flap valve, graded as Type III.

Following comprehensive patient consultation, the feasibility of endoscopic anti-reflux therapy was contemplated, subsequently leading to the application of hybrid argon plasma coagulation (hAPC) in the context of anti-reflux mucosal ablation ( Video 1 ). During the procedure, we initiated the delineation of the ablation territory employing hAPC, forming a horseshoe-shaped demarcation ( Fig. 2 **a** ). Subsequently, we executed submucosal injections directly within the marked region, utilizing hAPC ( Fig. 2 **b** ), followed by the argon plasma coagulation procedure atop the elevated mucosal surface ( Fig. 2 **c** ). Key technical nuances comprise the execution of ablation spanning a width of approximately 2–4 centimeters, with a circumferential dimension exceeding 90%, and reaching the submucosal layerʼs depth ( Fig. 2 **d** ). Postoperatively, the patient commenced a liquid diet after 24 hours; no perforation, hemorrhage, or pyrexia were encountered. One month post-procedure, mucosal flap valve reshaping was observed, resulting in the formation of an unobstructed contractile annulus ( Fig. 3 ).

**Fig. 2:**
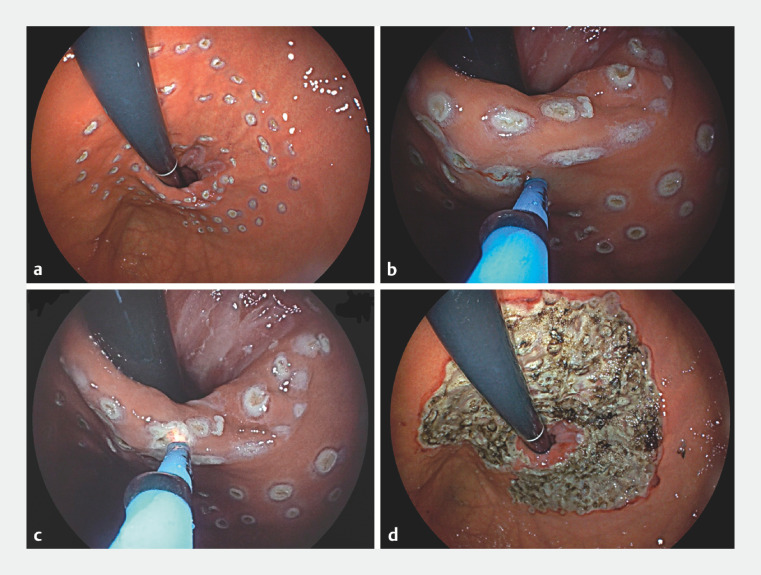
**a** A delineation of the ablation territory employing hybrid argon plasma coagulation (hAPC), forming a horseshoe-shaped demarcation. **b** Submucosal injections within the marked region, utilizing hAPC. **c** The argon plasma coagulation procedure atop the elevated mucosal surface. **d** The execution of ablation spanning a width of approximately 2–4 centimeters, with a circumferential dimension exceeding 90%, and reaching the submucosal layerʼs depth.

**Fig. 3:**
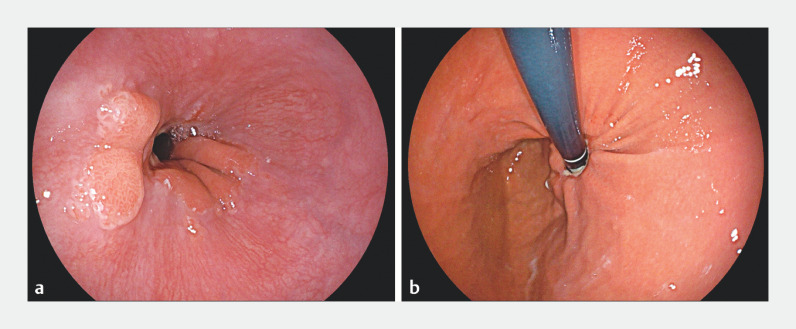
One month post-procedure, mucosal flap valve reshaping was observed, resulting in the formation of an unobstructed contractile annulus.

Hybrid argon plasma coagulation-assisted mucosal ablation in the management of refractory gastroesophageal reflux disease.Video 1

The application of anti-reflux mucosal ablation in the management of GERD 1 has been substantiated as an efficacious and secure approach, culminating in the amelioration of GERD-related symptoms and enhancement of overall quality of life 2 3 . A pioneering technique called hAPC amalgamates submucosal injections via a high-pressure water jet system, thereby establishing a protective cushion preceding ablation, ensuring that the ablation reaches the submucosal layer without incurring excessive harm 4 . Our utilization of hAPC in anti-reflux mucosal ablation not only attested to the achievement of submucosal ablation but also mitigated the inherent risks of perforation and postoperative stricture. To our knowledge, this represents the inaugural global case report delineating the implementation of hAPC within anti-reflux mucosal ablation for GERD management, thereby furnishing a salient reference for subsequent clinical therapeutic endeavors.

Endoscopy_UCTN_Code_TTT_1AO_2AJ

## Notes

Endoscopy E-Videos https://eref.thieme.de/e-videos
*E-Videos* is an open access online section of the journal *Endoscopy* , reporting on interesting cases and new techniques in gastroenterological endoscopy. All papers include a high-quality video and are published with a Creative Commons CC-BY license. Endoscopy E-Videos qualify for HINARI discounts and waivers and eligibility is automatically checked during the submission process. We grant 100% waivers to articles whose corresponding authors are based in Group A countries and 50% waivers to those who are based in Group B countries as classified by Research4Life (see: https://www.research4life.org/access/eligibility/ ). This section has its own submission website at https://mc.manuscriptcentral.com/e-videos .

## References

[1] Inoue H Tanabe M de Santiago E Anti-reflux mucosal ablation (ARMA) as a new treatment for gastroesophageal reflux refractory to proton pump inhibitors: a pilot study Endosc Int Open 2020 8 E133 E138 10.1055/a-1031-9436 32010745 PMC6976329

[2] Hernández Mondragón OV Zamarripa Mottú RA García Contreras LF Clinical feasibility of a new antireflux ablation therapy on gastroesophageal reflux disease (with video) Gastrointest Endosc 2020 92 1190 1201 32343977 10.1016/j.gie.2020.04.046

[3] Chou CK Chen CC Chen CC Positive and negative impact of anti-reflux mucosal intervention on gastroesophageal reflux disease Surg Endosc 2023 37 1060 1069 10.1007/s00464-022-09605-z 36109362

[4] Kolb JM Sagar S Anastasia C Hybrid argon plasma coagulation for Barrettʼs esophagus VideoGIE 2021 6 339 341 34401625 10.1016/j.vgie.2021.04.002 PMC8353141

